# Conventional and retrospective change in health-related quality of life of trauma patients: an explorative observational follow-up study

**DOI:** 10.1186/s12955-020-01404-1

**Published:** 2020-05-27

**Authors:** Juanita A. Haagsma, Inge Spronk, Mariska A. C. de Jongh, Gouke J. Bonsel, Suzanne Polinder

**Affiliations:** 1 Department of Public Health, Erasmus MC University Medical Center Rotterdam, P.O. Box 2040, 3000 CA Rotterdam, The Netherlands; 2 Association of Dutch Burn Centres, Maasstad Hospital, Rotterdam, The Netherlands; 3 Department Trauma TopCare, ETZ Hospital, Tilburg, The Netherlands

**Keywords:** Health-related quality of life, Mental recall, Wounds and injuries, Brain injuries traumatic

## Abstract

**Background:**

Within trauma care measurement of changes in health-related quality of life (HRQL) is used in understanding patterns of recovery over time. However, conventionally-measured change in HRQL may not always reflect the change in HRQL as perceived by the patient. Recall bias and response shift may contribute to disagreement between conventional and retrospective change in HRQL. This study aimed to measure conventional and retrospective change of HRQL and assess to which extent recall bias and response shift contribute to disagreement between these two in a heterogeneous sample of adult trauma patients.

**Methods:**

A sample of trauma patients (≥18 years) who attended the Emergency Department and were admitted to an Intensive Care unit or ward of one of ten Dutch hospitals received postal questionnaires 1 week (T1) and 3 months (T2) post-injury. At T1 and T2 participants completed the EQ-5D-3 L and EQ-VAS for their current health status. At T2 participants also filled out a recall and then-test regarding their health status at T1. The responses were used to assess conventional and retrospective change, recall bias and response shift. Wilcoxon signed rank tests were used to examine conventional and retrospective change on a group level. The intraclass correlation coefficient (ICC) was used to examine individual agreement between conventional and retrospective change. Uni- and multivariate linear regression analysis were used to investigate the association between background factors and recall bias and response shift.

**Results:**

The EQ-5D-3 L, recall and then-test were completed by 550 patients. Mean EQ-5D-3 L summary score improved from 0.48 at T1 to 0.74 at T2. Mean EQ-VAS score improved from 56 at T1 to 73 at T2. Retrospective change was significantly higher than conventional change (EQ-5D-3 L: Z = -5.2, *p* < 0.05; EQ-VAS Z = -2.1, *p* < 0.05). Pairwise comparisons showed that agreement between conventional and retrospective change was fair (EQ-5D-3 L: ICC = 0.49; EQ-VAS: ICC = 0.48). For EQ-5-3 L response shift was significantly higher than recall bias (Z = − 4.5, *p* < 0.05). Patients with traumatic brain injury (TBI), severe injury and/or posttraumatic stress symptoms were more susceptible to recall bias and response shift.

**Conclusions:**

We conclude that, compared to recall bias, response shift contributed more to the disagreement between conventional and retrospective change in EQ-5D-3 L summary score and EQ-VAS. Predictable subgroups of trauma patients were more susceptible to recall bias and response shift.

## Background

A well-studied outcome in trauma care is health-related quality of life (HRQL) [1]. HRQL is used to estimate the impact of an injury on a patient’s life, and enables to evaluate quality of care in patients [2]. Measurement of change in HRQL, individual or aggregate, has been used to evaluate health interventions in a wide range of conditions and populations (e.g. [3–7]). Inaccurate measurement of change in HRQL may therefore affect clinical practice and health care, and ultimately the quality of care and HRQOL of patients. In an observational context this change provides insight into patterns of recovery over time [8, 9]. The understanding of recovery patterns supports the clinician in setting expectations, and the timely identification of specific patient groups with lower HRQL over time. Knowing who faces a poor prognosis may guide the development and application of targeted interventions to halt this development.

However, conventionally-measured change in HRQL may not always reflect the change in HRQL as perceived or experienced by the patient. Conventionally-measured change in HRQL is defined by the difference between the direct measurements of HRQL at two consecutive occasions. The patient’s perceived change in HRQL is defined as the difference between the directly measured current HRQL and the HRQL as stated by the patient to be the HRQL on a specified previous occasion. Indeed, McPhail showed that agreement between conventional change and retrospective change in HRQL was not strong. A large proportion of the disagreement was attributed to so-called recall bias [10]. Recall bias is defined as a systematic measurement error, due to memory decay, that is the fading of memory with time. From the current standpoint, past health may be memorized as more deteriorated or better than it actually was; the direction depending on psychological mechanisms which keep better or worse memories better alive [11]. The magnitude of recall bias may depend on the scale that is used to measure HRQL, where subjective scales, such as the visual analogue scale (VAS), may easier be distorted than classification-like scales, like the EQ-5D [12, 13]. Recall bias of HRQL has been observed among patients with e.g. multiple sclerosis, psoriasis, cancer, injury and total hip arthroplasty [14–17] A study among patients with traumatic brain showed that recall bias was stronger in patients with high symptoms, which include memory problems [18]. This indicates that the size of recall bias may be higher among patients who experience memory problems compared to their counterparts.

Response shift may also contribute to disagreement between conventional and retrospective change in HRQL. Response shift is a true change of a patients’ perspective towards the targeted construct, caused by a change in internal standards, a change in values, and/or a redefinition of the construct [19, 20]. This may change the direction of change. Among trauma patients response shift may occur between multiple post-injury HRQL measurements due to patients adapting to their ill health [19, 20]. However, the magnitude of response shift may vary across type and severity of injury. A study among multiple sclerosis patients showed that being more disabled was associated with a change in internal standards with regards to certain HRQL dimensions [21], indicating that response shift may be stronger among patients with more severe injuries. Consequently, the contribution of response shift to disagreement between conventional and retrospective change may also vary across subgroups of trauma patients.

McPhail et al. were the first to investigate response shift and recall bias simultaneously in a sample of 101 elderly hospitalized patients [10]. The investigators argued that the contribution of response shift and recall bias may vary across other patient groups. This may particularly be the case for trauma patients, since injuries comprise of heterogeneous patterns of ill-health and may affect patients of all age groups.

The aims of this study were to measure conventional and retrospective change of HRQL, measured with the EQ-5D-3 L and the EQ-VAS, and to assess to which extent recall bias and response shift contribute to disagreement, in a heterogeneous sample of trauma patients.

### Hypotheses

We tested the following hypotheses:

- Agreement between conventional and retrospective change of HRQL, as measured with EQ-VAS, is lower compared to the agreement if HRQL was measured with EQ-5D-3 L, because recall bias and response shift more easily distort subjective scales (like a VAS) than a classification-like scale like EQ-5D-3 L.
- Agreement between conventional and retrospective change of HRQL is higher among trauma patients with less severe injuries (ISS < 16), because low impact trauma requires less adaptation to ones (final) health status compared severe trauma.
- Recall bias rather than response shift causes disagreement between conventional and retrospective change because with the time lapse chosen (3 months) memory problems affecting recall are no longer trivial.
- In older patients, in patients with traumatic brain injury (TBI) and patients with posttraumatic stress disorder (PTSD) the size of recall bias is higher compared to their counterparts, because these patients experience more memory problems.

## Methods

### Study design

This study utilizes data from a registry-based study on injury patients in Noord-Brabant (2.5 M inhabitants), the Netherlands. This prospective longitudinal cohort study, called the Brabant Injury Outcome Surveillance (BIOS) study, assessed outcomes in trauma patients, admitted to one of the ten hospitals in the Noord-Brabant region in the Netherlands [22]. The BIOS study includes multiple HRQL measurements up to 24 months after injury. Response shift items and recall questions were intentionally included in the 3 month follow-up survey. Ethical approval for the observational data analysis of this study was received from the Medical Ethics Committee Brabant (NL50258.028.14).

### BIOS study population

All trauma patients (≥18 years), who attended the Emergency Department (ED) and were admitted to an Intensive Care unit (ICU) or ward of one of the ten hospitals between November 2015 and November 2016, and who were discharged alive, qualified for inclusion. Patients were excluded if they were unable to understand or answer Dutch language questionnaires, when they had a pathological fracture due to a primary malignancy, or when they had no permanent address [22].

The eligible patients were invited to participate in the BIOS study via a postal invitation one week after admission to hospital. This invitation was accompanied by an informed consent form and the first survey (T1). For this procedure ethical permission is obtained. Non-responders received a telephone call to discuss their participation. The 3 month follow up survey (T2) was sent to the patient if consent and the completed T1 survey were received by the researchers. For present study, we included data from patients who completed both surveys.

### Self-report measures

T1 included questions on patient characteristics (e.g. age and gender), and 19 items regarding the presence of one or more chronic diseases (e.g. diabetes) prior to the injury to assess comorbidity [23]. If a patient suffered from one or more chronic disease(s) additional to the injury that qualified for inclusion, he/she was defined as having comorbidity [24]. Level of education was divided in three categories: low, middle or high. For patients classified as low level the highest level of education obtained was no education, primary school or prevocational education. Patients classified as middle level followed at best secondary or vocational education, and patients classified as high completed professional higher education or university level. Both surveys (T1, T2) included the EQ-5D-3 L.

The EQ-5D-3 L is a standardized generic HRQL measure [25]. The EQ-5D-3 L covers five dimensions: mobility, self-care, usual activities, pain/discomfort, and anxiety/depression and a visual analogue scale (EQ-VAS) [25]. The five dimensions have three response options: no problems, moderate problems, extreme problems [26]. The ordinal scores on the dimensions can be used in a descriptive analysis, but may also be used as an input to calculate an EQ-5D-3 L summary score combining all dimensions, ranging from 0 (death) and 1 (full health) [27]. For few health states considered worse than death the summary score can have a value lower than zero. The EQ-VAS consists of a scale from 0 (worst imaginable health) to 100 (best imaginable health) and measures the patient’s self-rated health in a subjective way. Apart from the complete EQ-5D-3 L, the T2 questionnaire also included the so-called ‘recall’ and the ‘then’ test. The recall test asked patients to report what they remember to have reported on the EQ-5D-3 L on the previous occasion (T1). The then test asked patients to report how they believe now what their health status was at previous assessment (T1). Both the recall test and then test consisted of six items: five EQ-5D-3 L items and the EQ-VAS.

The T2 survey also included the impact of event scale (IES) [28]. The IES is a validated self-report instrument that uses 15-items questionnaire to assess stress symptoms caused by a traumatic event. Each item is scored on a 4-point scale (0, 1, 3, 5 points), where 0 refers to “not at all” and 5 refers to “extremely”. The total IES-score ranges from 0 (no meaningful impact into any direction) to 75 (severe impact event on all 15 items). PTSD is assumed to be present if IES-score exceeds 35 [29].

### Injury data

Apart from the self-report data, clinical injury data of included trauma patients were available from the Brabant Trauma Registry. All BIOS hospitals also participate in this registry. Injury data comprised the Injury Severity Score (ISS) [30] and the Abbreviated Injury Scale (AIS) [31]. The AIS classifies the severity of a trauma via an anatomic scale and it scores the type, location and severity of each injury that was sustained by a patient. The AIS score of the three most severely injured body regions are squared and summed to an ISS. The ISS is an accepted summary score for the severity of a trauma, and ranges from 1 to 75. A major trauma is assumed to be present if the ISS exceeds 15 [32]. The ISS was automatically calculated based on the AIS scores that were registered in the Brabant Trauma Registry.

### Data analysis

SPSS version 23 was used for all analyses. We performed a non-response analysis to study whether responders differed from non-responders. Mann Whitney U tests were used for continuous variables and Chi-square tests for categorical variables. Descriptive statistics were used to assess the sample characteristics, and EQ-5D-3 L dimension, EQ-5D-3 L summary scores and EQ-VAS scores.

T1 health outcomes were compared between subgroups: males vs. females, age < 65 vs. ≥65 years, absence vs. presence of pre-existing comorbidity, absence vs. presence of traumatic brain injury (TBI), ISS < 16 vs. ISS ≥ 16, and absence vs. presence of PTSD using Mann Whitney U tests. Similarly, Kruskal Wallis test was used to compare outcomes according to educational attainment (three levels).

The following equations were used to calculate conventional and retrospective change in HRQL:

1 $$ {\mathrm{Conventional}\ \mathrm{change}}_{\mathrm{EQ}-5\mathrm{D}\ \mathrm{summary}\ \mathrm{score}\ \mathrm{T}1,\mathrm{T}2}=\mathrm{EQ}-5\mathrm{D}-3\;\mathrm{L}\ {\mathrm{summary}\ \mathrm{score}}_{\mathrm{T}2}-\mathrm{EQ}-5\mathrm{D}-3\;\mathrm{L}\ {\mathrm{summary}\ \mathrm{score}}_{\mathrm{T}1} $$

Where EQ-5D-3 L summary score_T2_ and EQ-5D-3 L summary score_T1_ are the directly measured EQ-5D-3 L at T1 and T2, respectively.

2 $$ {\mathrm{Conventional}\ \mathrm{change}}_{\mathrm{EQ}-\mathrm{VAS}\ \mathrm{T}1,\mathrm{T}2}=\mathrm{EQ}-{\mathrm{VAS}}_{\mathrm{T}2}-\mathrm{EQ}-{\mathrm{VAS}}_{\mathrm{T}1} $$

Where EQ-VAS_T2_ and EQ-VAS_T1_ are the directly measured EQ-VAS scores at T1 and T2, respectively.

3 $$ {\mathrm{Retrospective}\ \mathrm{change}}_{\mathrm{EQ}-5\mathrm{D}\ \mathrm{summary}\ \mathrm{score}\ \mathrm{T}1,\mathrm{T}2}=\mathrm{EQ}-5\mathrm{D}-3\;\mathrm{L}\ {\mathrm{summary}\ \mathrm{score}}_{\mathrm{T}2}-\mathrm{EQ}-5\mathrm{D}-3\;\mathrm{L}\ \mathrm{then}\ \mathrm{test} $$

Where EQ-5D-3 L summary score_T2_ is the directly measured EQ-5D-3 L at T2 and EQ-5D-3 L then test is the EQ-5D-3 L summary score of how the respondents believed their EQ-5D-3 L health status was at previous assessment (T1).

4 $$ {\mathrm{Retrospective}\ \mathrm{change}}_{\mathrm{EQ}-\mathrm{VAS}\ \mathrm{T}1,\mathrm{T}2}=\mathrm{EQ}-{\mathrm{VAS}}_{\mathrm{T}2}-\mathrm{EQ}-\mathrm{VAS}\ \mathrm{then}\ \mathrm{test} $$

Where EQ-VAS_T2_ is the directly measured EQ-VAS score at T2 and EQ-VAS then test is the EQ-VAS score of how the respondents believed their EQ-VAS score was at previous assessment (T1).

**Fig. 1:**
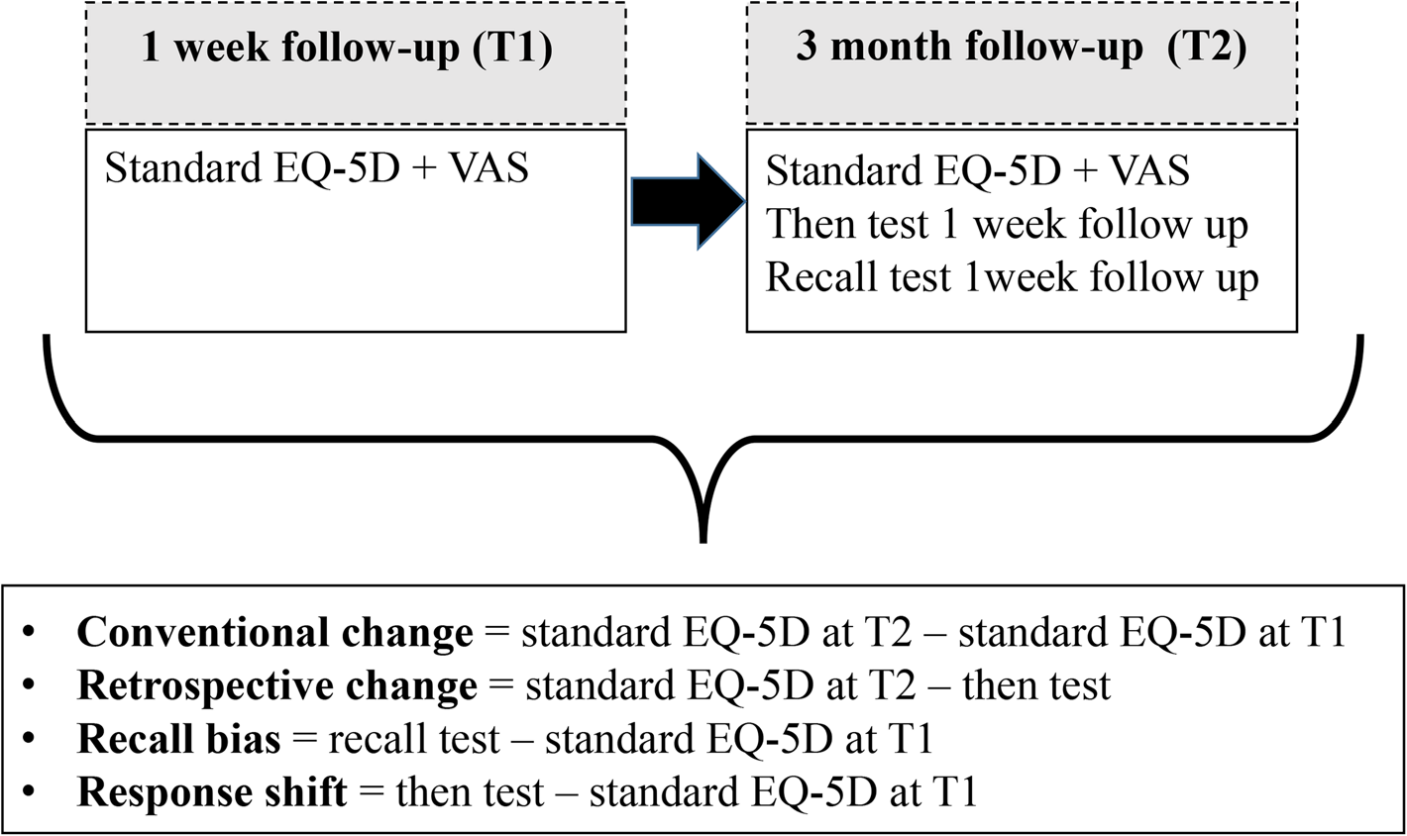
Schematic overview of the calculations of conventional and retrospective change, recall bias and response shift

See Fig. 1 for a schematic overview of the calculations of conventional and retrospective change, recall bias and response shift.

Wilcoxon signed rank tests were used to compare conventional and retrospective change in EQ-5D-3 L summary scores and EQ-VAS scores for the total sample as well as for subgroups (males vs. females, age < 65 vs. ≥65 years, absence vs. presence of pre-existing comorbidity, absence vs. presence of traumatic brain injury (TBI), ISS < 16 vs. ISS ≥ 16, and absence vs. presence of PTSD). It was examined whether conventional and retrospective change in EQ-5D-3 L summary scores and EQ-VAS scores differed for the different subgroups, and whether this was in accordance to our hypotheses. We calculated the intraclass correlation coefficient (ICC) to assess agreement between conventional and retrospective change on patient level [33]. We calculated the ICC for the whole group, and for subgroups (age, gender, educational level, comorbidity status, ISS category and PTSD symptoms). ICC was defined as poor (< 0.40), fair (0.40–0.59), good (0.60–0.74) or excellent (0.75–1.00) [34].

Recall bias and response shift were calculated with the following equations:

5 $$ {\mathrm{Recall}\ \mathrm{bias}}_{\mathrm{EQ}-5\mathrm{D}\ \mathrm{summary}\ \mathrm{score}}=\mathrm{EQ}-5\mathrm{D}-3\;\mathrm{L}\ \mathrm{recall}\ \mathrm{test}-\mathrm{EQ}-5\mathrm{D}-3\;\mathrm{L}\ {\mathrm{summary}\ \mathrm{score}}_{\mathrm{T}1} $$

Where EQ-5D-3 L recall test is the EQ-5D-3 L summary score of the EQ-5D-3 L health status that the respondents remember to have reported at the previous assessment (T1) and EQ-5D-3 L summary score_T1_ is the directly measured EQ-5D-3 L at T1.

6 $$ {\mathrm{Recall}\ \mathrm{bias}}_{\mathrm{EQ}-\mathrm{VAS}}=\mathrm{EQ}-\mathrm{VAS}\ \mathrm{recall}\ \mathrm{test}-\mathrm{EQ}-{\mathrm{VAS}}_{\mathrm{T}1} $$

Where EQ-VAS recall test is the EQ-VAS score that the respondents remember to have reported at the previous assessment (T1) and EQ-VAS_T1_ is the directly measured EQ-VAS at T1.

7 $$ {\mathrm{Response}\ \mathrm{shift}}_{\mathrm{EQ}-5\mathrm{D}\ \mathrm{summary}\ \mathrm{score}}=\mathrm{EQ}-5\mathrm{D}-3\;\mathrm{L}\ \mathrm{then}\ \mathrm{test}-\mathrm{EQ}-5\mathrm{D}-3\;\mathrm{L}\ {\mathrm{summary}\ \mathrm{score}}_{\mathrm{T}1} $$

Where EQ-5D-3 L then test is the EQ-5D-3 L summary score of how the respondents believed their EQ-5D-3 L health status was at previous assessment (T1) and EQ-5D-3 L summary score_T1_ is the directly measured EQ-5D-3 L at T1.

8 $$ {\mathrm{Response}\ \mathrm{shift}}_{\mathrm{EQ}-\mathrm{VAS}}=\mathrm{EQ}-\mathrm{VAS}\ \mathrm{then}\ \mathrm{test}-\mathrm{EQ}-{\mathrm{VAS}}_{\mathrm{T}1} $$

Where EQ-VAS then test is the EQ-VAS score of how the respondents believed their health status was at previous assessment (T1) and EQ-VAS_T1_ is the directly measured EQ-VAS at T1.

Wilcoxon signed rank tests were used to examine the differences between response shift and recall bias. Differenced were also studied on a subgroup level in order to examine whether recall bias and response shift differs between the subgroups defined. To estimate the role of background factors in recall bias and response shift respectively, we predicted recall bias and response shift from the socio-demographic factors (age, gender, education), TBI (yes or no), injury severity level (ISS as a continuous variable) and PTSD symptoms (IES-score as a continuous variable). Straightforward univariate and multivariate linear regression analysis were applied, with backward selection (deselection criterion *p* < 0.10) were used to investigate the association between socio-demographics, comorbidity, TBI, injury severity, PTSD and recall bias and response shift.

Overall *p*-values< 0.05 were considered to indicate statistical significance, although our analysis primarily was explorative.

## Results

### Study population

In total, 1518 of the 5731 invited patients participated in the BIOS study (26.5%). Responders were significantly younger than non-responders (*p* < 0.05) and significantly more often male than non-respondents (*p* < 0.05). In total, 790 patients responded on the T1 survey and 1351 patients responded on the T2 survey. However, only 550 of these patients completed the EQ-5D-3 L and EQ-VAS at T1 and T2, the then-test EQ-5D-3 L and EQ-VAS (at T2) and the recall EQ-5D-3 L and EQ-VAS (at T2) and were therefore included in this study. These 550 completers were significantly more often male, significantly younger, higher educated and had a shorter hospital stay compared to non-completer. The completers had a mean age of 61.0 years (SD 16.0) and slightly more than half of the participants (56.0%) was male (Table 1). Most participants had a middle or high-level education and comorbidity was highly prevalent (56.2%). Patients’ median hospital stay was 4.0 (IQR 2.0–6.0) days, and most common injuries were mild traumatic brain injury (28.9%) and hip fracture (20.7%). Median ISS was 5.0 (IQR 4.0–9.0).

**Table 1:** Characteristics of study population

**Characteristic**	**Study population (*n*** **= 550)**
**Gender:** Male	308 (56.0%)
**Age** (Mean, SD)	61.0 (16.0)
**Education**	
Low	127 (23.1%)
Middle	214 (38.9%)
High	199 (36.2%)
Unknown	10 (1.8%)
**Comorbidity status**	
No comorbidity	231 (42.0%)
Comorbidity	309 (56.2%)
Unknown	10 (1.8%)
**Length of hospital stay in days (Median, IQR)**	4.0 (2.0–6.0)
**Number of injuries**	
1	275 (50.0%)
2	129 (23.5%)
≥ 3	146 (26.5%)
**Type of injury**^**b**^	
Mild TBI	159 (28.9%)
Hip fracture	114 (20.7%)
Pelvic injury	71 (12.9%)
Rib fracture	71 (12.9%)
Tibia, complex foot or femur fracture	71 (12.9%)
Shoulder and upper arm injury	57 (10.4%)
Stable vertebral fracture or disc injury	40 (7.3%)
Radius, ulna or hand fracture	40 (7.3%)
Thoracic injury	31 (5.6%)
Facial fracture	29 (5.3%)
Severe TBI	15 (2.7%)
Mild abdominal injury	14 (2.5%)
Severe abdominal injury	5 (0.9%)
Spinal cord injury	2 (0.4%)
**Injury Severity Score**	
< 8	320 (58.2%)
8–16	193 (35.1%)
≥ 16	35 (6.4%)
Unknown	2 (0.4%)
**PTSD symptoms**^**a**^	
No PTSD (IES < 35)	451 (82.0%)
PTSD (IES ≥ 35)	41 (7.5%)
Unknown	58 (10.5%)
**EQ-5D-3 L scores at T1**	
Utility score (Mean, SD)	0.482 (0.298)
EQ-VAS (Mean, SD)	56.3 (20.5)
Mobility (% reporting moderate or extreme problems)	72.0%
Self-care (% reporting moderate or extreme problems)	69.8%
Usual activities (% reporting moderate or extreme problems)	85.5%
Pain/discomfort (% reporting moderate or extreme problems)	88.0%
Anxiety/depression (% reporting moderate or extreme problems)	24.9%

Note. *SD* standard deviation, *IQR* inter quartile range

^a^PTSD symptoms measured with the Impact of Event Scale (IES) 3 months post-injury

^b^ The total number of patients by type of injury exceeds 550, because many respondents had multiple injuries

### EQ-5D-3 L – conventional change versus retrospective change

Table 2 shows the mean EQ-5D-3 L summary score at T1 and T2, mean Then Test, mean conventional change and retrospective change between EQ-5D-3 L summary scores at T1 and T2. Mean EQ-5D-3 L summary scores at T1 and T2 were 0.482 (SD 0.30) and 0.735 (SD 0.24), respectively. A lower EQ-5D-3 L summary score at T1 was associated with being younger, having an ISS ≥ 16, not having a TBI and having PTSD three months post-injury (all *p* < 0.05). Pairwise comparisons showed that agreement between retrospective and conventional change was fair (ICC = 0.49, *p* < 0.05) (see Table 2). Retrospective change was significantly higher compared to conventional change (Z = -5.2, *p* < 0.05). The difference between conventional and retrospective change was highest among patients with ISS ≥ 16 (mean difference = − 0.12, Z = -1.9, *p* = 0.058).

**Table 2:** Mean EQ-5D-3 L summary score at T1, conventional change and retrospective change between EQ-5D-3 L summary scores at T1 and T2 and magnitude of recall bias and response shift

	**n**	**Mean EQ-5D T1**	**Mean EQ-5D T2**	**Mean Then Test**	**Conventional change**	**Retrospective change**	**ICC**^**a**^	**Mean Recall Test**	**Recall bias**	**Response shift**	**ICC**^**b**^
**Total**	550^c^	0.482 (SD 0.30)	0.735 (SD 0.24)	0.419 (SD 0.35)	0.254 (SD 0.29)	0.316 (SD 0.31)	0.489^*^	0.462 (SD 0.34)	−0.020 (SD 0.30)	− 0.063 (SD 0.31)	0.681^*^
**Gender**											
Males	308	0.500 (SD 0.31)	0.741 (SD 0.25)	0.450 (SD 0.36)	0.242 (SD 0.31)	0.292 (SD 0.33)	0.545^*^	0.506 (SD 0.35)	0.007 (SD 0.31)	−0.050 (SD 0.30)	0.705^*^
Females	242	0.459 (SD 0.28)	0.728 (SD 0.23)	0.380 (SD 0.33)	0.269 (0.26)	0.348 (SD 0.30)	0.391^*^	0.405 (SD 0.33)	−0.054 (SD 0.29)	− 0.079 (SD 0.31)	0.648^*^
**Age**											
< 65 years	285	0.452 (SD 0.29)	0.741 (SD 0.24)	0.373 (SD 0.34)	0.289 (SD 0.29)	0.369 (SD 0.32)	0.516^*^	0.425 (SD 0.34)	−0.028 (SD 0.31)	− 0.080 (SD 0.30)	0.749^*^
65+ years	265	0.513 (SD 0.30)	0.729 (SD 0.24)	0.469 (SD 0.35)	0.216 (SD 0.29)	0.260 (SD 0.30)	0.435^*^	0.501 (SD 0.35)	−0.012 (SD 0.29)	− 0.044 (SD 0.31)	0.608^*^
**Educational level**^**1**^											
Low	127	0.503 (SD 0.33)	0.697 (SD 0.26)	0.443 (SD 0.37)	0.193 (SD 0.30)	0.254 (SD 0.30)	0.439^*^	0.489 (SD 0.35)	−0.015 (SD 0.32)	− 0.060 (SD 0.32)	0.661^*^
Middle	214	0.492 (SD 0.30)	0.740 (SD 0.25)	0.406 (SD 0.350)	0.248 (SD 0.30)	0.334 (SD 0.30)	0.458^*^	0.451 (SD 0.36)	−0.041 (SD 0.32)	−0.086 (SD 0.31)	0.717^*^
High	199	0.447 (SD 0.27)	0.752 (SD 0.23)	0.403 (SD 0.34)	0.305 (SD 0.26)	0.349 (SD 0.33)	0.543^*^	0.448 (SD 0.33)	0.012 (SD 0.26)	−0.044 (SD 0.28)	0.650^*^
**Comorbidity status**^**2**^											
No comorbidity	231	0.499 (SD 0.28)	0.784 (SD 0.24)	0.437 (SD 0.33)	0.285 (SD 0.28)	0.347 (SD 0.31)	0.532^*^	0.480 (SD 0.32)	−0.019 (SD 0.30)	−0.062 (SD 0.29)	0.736^*^
Comorbidity	309	0.472 (SD 0.31)	0.702 (SD 0.24)	0.407 (SD 0.36)	0.230 (SD 0.29)	0.295 (SD 0.32)	0.450^*^	0.452 (SD 0.36)	−0.020 (SD 0.30)	−0.065 (SD 0.32)	0.655^*^
**ISS**^**3**^											
ISS < 16	513	0.493 (SD 0.29)	0.741 (SD 0.24)	0.433 (SD 0.34)	0.248 (SD 0.29)	0.308 (SD 0.31)	0.503^*^	0.474 (SD 0.34)	−0.019 (SD 0.30)	−0.060 (SD 0.30)	0.670^*^
ISS > = 16	35	0.350 (SD 0.30)	0.659 (SD 0.28)	0.229 (SD 0.40)	0.309 (SD 0.29)	0.429 (SD 0.36)	0.315^*^	0.289 (SD 0.38)	−0.061 (SD 0.34)	−0.120 (SD 0.39)	0.740^*^
**TBI**											
No TBI	384	0.432 (SD 0.28)	0.721 (SD 0.24)	0.383 (SD 0.33)	0.290 (SD 0.28)	0.338 (SD 0.31)	0.461^*^	0.428 (SD 0.33)	−0.004 (SD 0.30)	−0.048 (SD 0.31)	0.656^*^
TBI	166	0.598 (SD 0.30)	0.768 (SD 0.25)	0.501 (SD 0.38)	0.171 (SD 0.28)	0.267 (SD 0.31)	0.525^*^	0.539 (SD 0.37)	−0.058 (SD 0.29)	−0.096 (SD 0.29)	0.742^*^
**PTSD symptoms**^**4d**^											
No PTSD	451	0.490 (SD 0.29)	0.759 (SD 0.22)	0.423 (SD 0.34)	0.269 (SD 0.28)	0.337 (SD 0.31)	0.518^*^	0.471 (SD 0.34)	−0.019 (SD 0.30)	−0.068 (SD 0.29)	0.684^*^
PTSD	41	0.345 (SD 0.35)	0.487 (SD 0.35)	0.270 (SD 0.43)	0.142 (SD 0.41)	0.216 (SD 0.35)	0.294^*^	0.252 (SD 0.36)	−0.093 (SD 0.37)	− 0.075 (SD 0.45)	0.780^*^

*SD* standard deviation, *ICC* intraclass correlation coefficient, *ISS* injury severity score, *TBI* traumatic brain injury, *PTSD* posttraumatic stress disorder

^a^ ICC corresponds to the correlation between conventional and retrospective change

^b^ ICC corresponds to the correlation between recall bias and response shift

^c^Patients who completed the EQ-5D at 1 week and 3 months and the then-test and recall-test for 1 week at 3 months after sustaining an injury

^d^PTSD symptoms measured with the Impact of Event Scale (IES) 3 months post-injury

^1^10 missing values, ^2^10 missing values, ^3^2 missing values, ^4^58 missing values

^*^p < 0.05

### EQ-5D-3 L – recall bias versus response shift

Recall bias and response shift are also shown in Table 2. Average recall bias ranged from − 0.09 (patients with PTSD) to − 0.004 (patients with TBI). Overall, recalled T1 EQ-5D-3 L was lower (− 0.02) than the directly assessed EQ-5D-3 L, except for males and patients with a high educational level (all *p* < 0.05). Pairwise comparisons showed that agreement between recall bias and response shift was good (ICC = 0.68, *p* < 0.05). Multivariate linear regression analysis indicated that increasing PTSD symptoms were associated with recalling T1 EQ-5D-3 L as lower (‘worse’) than directly assessed EQ-5D-3 L at T1 (see Table 3). The EQ-5D-3 L dimensions that differed most frequently between the directly assessed EQ-5D-3 L at T1 and the recall test were usual activities (36.5% of the respondents chose a different response option on the recall test), pain and/or other complaints (34.4%), self-care (28.7%) and anxiety/depression (27.5%).

**Table 3:** Multivariate models for recall bias on the EQ-5D-3 L summary score

	**Initial model**		**Final model**	
	**Unstandardized B**	***p*-value**	**Unstandardized B**	***p*-value**
Constant	0.070	0.342	0.009	0.603
Age^a^	0.000	0.769		
Sex (male/female)	−0.044	0.119		
Education (dichotomized)	0.025	0.379		
Comorbidity (no/yes)	0.031	0.312		
TBI (no/yes)	−0.051	0.093		
ISS^a^	0.001	0.764		
PTSD^a^	−0.003	0.001	−0.004	< 0.001
F value	2.96	0.05	13.73	< 0.001
Adjusted R-square	0.028		0.026	

^a^ Continuous variables

Mean response shift ranged from − 0.12 (patients with an ISS ≥ 16) to − 0.04 (patients with a high educational level and patients older than 65 years). Multivariate linear regression analysis indicated that increasing symptoms of PTSD were significantly associated with an increase in response shift (see Table 4). The EQ-5D-3 L dimensions that differed most frequently between the directly assessed EQ-5D-3 L at T1 and the then test that was used to assess response shift were pain and/or other complaints (35.6% of the respondents chose a different pain and/or other complaints response option on the then test), daily activities (34.5%), self-care (31.3%) and anxiety/depression (27.3%). Response shift, with an average value of − 0.06, was significantly higher than recall bias (Z = − 4.5, *p* < 0.05).

**Table 4:** Multivariate models for response shift based on the EQ-5D-3 L summary score

	**Initial model**		**Final model**	
	**Unstandardized B**	***p*-value**	**Unstandardized B**	***p*-value**
Constant	− 0.039	0.603	−0.049	
Age^a^	< 0.001	0.750		
Sex (male/female)	−0.025	0.387		
Education (dichotomized)	0.037	0.201		
Comorbidity (no/yes)	0.002	0.945		
TBI (no/yes)	−0.039	0.200		
ISS^a^	0.000	0.983		
PTSD^a^	−0.002	0.037	−0.002	0.016
F value	1.40	0.20	5.83	0.016
Adjusted R-square	0.006		0.010	

^a^ Continuous variables

### EQ-VAS – conventional change versus retrospective change

Table 5 shows the mean EQ-VAS score at T1 and the mean conventional change and retrospective change between EQ-VAS scores at T1 and T2. Mean EQ-VAS score improved from T1 (56.3; SD 20) to T2 (72.6; SD 17). A lower EQ-VAS score at T1 was associated with female gender, younger age, having an ISS ≥ 16 and not having a TBI (all *p* < 0.05). Individual agreement between retrospective and conventional change in EQ-VAS was fair (ICC = 0.483, *p* < 0.05) (see Table 5). Retrospective change in EQ-VAS score was significantly higher compared to conventional change (Z = -2.1, *p* < 0.05). The difference between conventional and retrospective change in EQ-VAS was particularly large among patients with PTSD (difference = − 7.7, Z = -2.4, *p* < 0.05), patients with an ISS ≥ 16 (mean difference = − 6.6, Z = -1.7, *p* = 0.09) and patients with a TBI (mean difference = − 4.7, Z = -2.9, *p* < 0.05).

**Table 5:** Mean EQ-VAS score at T1, conventional change and retrospective change between EQ-VAS score at T1 and T2 and magnitude of recall bias and response shift

	**EQ-VAS T1**	**Mean EQ-5D T2**	**Mean Then Test**	**Conventional change**	**Retrospective change**	**ICC**^**α**^	**Mean Recall Test**	**Recall bias**	**Response shift**	**ICC**^**§**^
**Total**	56.3^$^ (SD 20)	72.6 (SD 17.0)	54.7 (SD 21.0)	16.3 (SD 19)	17.9 (SD 18)	0.483^*^	55.7 (SD 21.0)	−0.62 (SD 20)	−1.59 (SD 19)	0.783^*^
**Gender**										
Males	58.3 (SD 21)	73.4 (SD 17.0)	56.4 (SD 22.5)	15.0 (SD 12)	17.0 (SD 14)	0.464^*^	57.6 (SD 22.5)	−0.74 (SD 21)	−1.94 (SD 19)	0.793^*^
Females	53.8 (SD 19)	71.7 (SD 16.9)	52.7 (SD 18.8)	17.9 (SD 19)	19.0 (SD 18)	0.503^*^	53.3 (SD 18.7)	−0.46 (SD 17)	−1.14 (SD 19)	0.768^*^
**Age**										
< 65 years	54.1 (SD 20)	72.1 (SD 17.9)	51.4 (SD 21.2)	17.9 (SD 19)	20.7 (SD 19)	0.459^*^	51.7 (SD 20.9)	−2.46 (SD 18)	−2.74 (SD 20)	0.740^*^
65+ years	58.7 (SD 21)	73.2 (SD 15.9)	58.3 (SD 20.2)	14.5 (SD 20)	14.8 (SD 16)	0.498^*^	60.0 (SD 20.2)	1.362 (SD 21)	−0.34 (SD 18)	0.825^*^
**Educational level**^**1**^										
Low	56.1 (SD 21)	69.7 (SD 16.9)	54.6 (SD 19.6)	13.6 (SD 21)	15.1 (SD 19)	0.561^*^	55.8 (SD 20.7)	−0.32 (SD 20)	−1.51 (SD 19)	0.798^*^
Middle	57.4 (SD 21)	74.3 (SD 17.2)	54.8 (SD 22.7)	17.0 (SD 20)	19.6 (SD 18)	0.402^*^	55.7 (SD 22.5)	−1.71 (SD 21)	−2.61 (SD 21)	0.773^*^
High	55.1 (SD 20)	72.8 (SD 16.3)	54.3 (SD 20.0)	17.7 (SD 17)	18.5 (SD 16)	0.519^*^	55.4 (SD 19.6)	0.32 (SD 18)	−0.80 (SD 16)	0.784^*^
**Comorbidity status**^**2**^										
No comorbidity	57.3 (SD 20)	77.0 (SD 16.3)	56.2 (SD 20.9)	19.7 (SD 20)	20.8 (SD 19)	0.524^*^	56.7 (SD 20.5)	−0.58 (SD 20)	−1.06 (SD 19)	0.772^*^
Comorbidity	55.6 (SD 20)	69.6 (SD 16.6)	53.9 (SD 20.8)	14.1 (SD 19)	15.7 (SD 17)	0.421^*^	55.3 (SD 21.1)	−0.27 (SD 20)	−1.676 (SD 19)	0.793^*^
**ISS**^**3**^										
ISS < 16	57.3 (SD 20)	73.1 (SD 16.8)	55.9 (SD 20.3)	15.8 (SD 19)	17.11 (SD 17)	0.503^*^	56.6 (SD 20.4)	−0.69 (SD 19)	−1.31 (SD 19)	0.802^*^
ISS > = 16	44.6 (SD 20)	66.5 (SD 18.3)	37.9 (SD 23.6)	21.9 (SD 23)	28.5 (SD 19)	0.199	43.8 (SD 25.3)	−0.77 (SD 29)	−6.66 (SD 27)	0.661^*^
**TBI**										
No TBI	54.5 (SD 20)	71.8 (SD 17.0)	54.3 (SD 20.6)	17.3 (SD 20)	17.5 (SD 18)	0.503^*^	55.1 (SD 20.5)	0.57 (SD 20)	−0.22 (SD 19)	0.787^*^
TBI	60.5 (SD 21)	74.4 (SD 16.7)	55.8 (SD 22.1)	13.9 (SD 17)	18.6 (SD 17)	0.445^*^	57.2 (SD 22.2)	−3.37 (SD 19)	−4.75 (SD 18)	0.765^*^
**PTSD symptoms**^**4&**^										
No PTSD	56.9 (SD 20)	73.7 (SD 16.3)	55.6 (SD 21.1)	16.8 (SD 19)	18.2 (SD 18)	0.495^*^	56.2 (SD 20.8)	−0.72 (SD 19)	−1.39 (SD 18)	0.774^*^
PTSD	51.8 (SD 21)	58.1 (SD 17.7)	44.2 (SD 20.8)	6.2 (SD 22)	13.9 (SD 19)	0.556^*^	44.5 (SD 21.2)	−7.29 (SD 20)	−7.61 (SD 19)	0.753^*^

*SD* standard deviation, *ICC* intraclass correlation coefficient, *ISS* injury severity score, *TBI* traumatic brain injury, *PTSD* posttraumatic stress disorder

^α^ ICC corresponds to the correlation between conventional and retrospective change

^§^ ICC corresponds to the correlation between recall bias and response shift

^$^Patients who completed the EQ-5D at 1 week and 3 months and the then-test and recall-test for 1 week at 3 months after sustaining an injury

^&^PTSD symptoms measured with the Impact of Event Scale (IES) 3 months post-injury

^1^10 missing values, ^2^10 missing values, ^3^2 missing values, ^4^58 missing values

### EQ-VAS – recall bias versus response shift

On average, the recalled T1 EQ-VAS was 0.6 lower (‘worse’) than directly assessed T1 EQ-VAS (*p* < 0.05). The mean recall bias ranged from − 7.3 for patients with PTSD to − 0.3 for patients with comorbidity. Overall, recalled T1 EQ-5D-3 L was lower than the directly assessed EQ-5D-3 L, except for patients aged 65 and older, patients without TBI and patients with a high educational level (all *p* < 0.05). Pairwise comparisons showed that agreement between recall bias and response shift was excellent (ICC = 0.78, *p* < 0.05). Multivariate linear regression analysis indicated that increasing PTSD symptoms and having TBI was significantly associated with a lower recalled T1 EQ-VAS compared to directly assessed EQ-VAS (see Table 6). With an average value of − 1.6, response shift was higher than recall bias, but this difference was not significant (Z = -0.635, *p* = 0.53). Response shift was highest for patients with PTSD (mean: − 7.6), patients with an ISS ≥ 16 (mean: − 6.7) and patients with TBI (mean: − 4.8) and lowest for patients without TBI (mean: − 0.2) and patients older than 65 years (mean: − 0.3). Multivariate linear regression analysis indicated that increasing PTSD symptoms and having TBI was associated with response shift (see Table 7).

**Table 6:** Multivariate models for response shift based on the EQ-VAS

	**Initial model**		**Final mode**l	
	**Unstandardized B**	***p*-value**	**Unstandardized B**	***p*-value**
Constant	−5.977	0.342	0.482	0.674
Age^a^	0.059	0.769		
Sex (male/female)	2.146	0.119		
Education (dichotomized)	1.482	0.379		
Comorbidity (no/yes)	0.351	0.312		
TBI (no/yes)	−3.922	0.093	−4.037	0.032
ISS^a^	−0.138	0.764		
PTSD^a^	−0.142	0.001	−0.135	0.029
F value	2.15	0.038	5.09	0.007
Adjusted R-square	0.017		0.017	

^a^ Continuous variables

**Table 7:** Multivariate models for recall bias based on the EQ-VAS

	**Initial model**		**Final model**	
	**Unstandardized B**	***p*-value**	**Unstandardized B**	***p*-value**
Constant	−8.476	0.069	1.127	0.333
Age^a^	0.083	0.178		
Sex	1.727	0.331		
Education (dichotomized)	1.889	0.299		
Comorbidity (yes/no)	1.400	0.474		
TBI (yes/no)	−3.685	0.055	−3.808	0.047
ISS^a^	0.100	0.521		
PTSD^a^	−0.147	0.022	−0.134	0.033
F value	2.16	0.036	4.62	0.010
Adjusted R-square	0.017		0.015	

^a^ Continuous variables

## Discussion

Our study showed that retrospective change in HRQL exceeded conventional change and that, at the individual level, agreement between conventional and retrospective change was only fair for both the EQ-5D-3 L summary score and the EQ-VAS. Response shift, more than recall bias, modified the reported retrospective outcome.

The relative magnitude of recall bias and response shift was higher when measured with the EQ-VAS compared to the EQ-5D-3 L summary score. This may be due to the fact that the restricted range of responses of the EQ-5D-3 L may lead to smaller variability in scores compared to the continuous EQ-VAS [13]. The subjectivity of the scale, which is higher for VAS compared to the classification-like EQ-5D-3 L, played a much smaller role than expected. We expected that the individual agreement between conventional and retrospective change would be higher for the EQ-5D-3 L summary score compared to the EQ-VAS; however, our findings showed that the individual agreement was similar.

In agreement to our expectations, response shift was higher among trauma patients with severe injuries (ISS ≥ 16). This indicates that high impact trauma requires more adaptation to one’s health status compared to less severe trauma. Our findings also showed that, relatively shortly after sustaining injury, the magnitude of response shift is already quite large. This is in agreement with a study that assessed response shift among individuals with stroke and that found evidence of similarly large magnitude of response shift 24 weeks post-stroke [35].

Conversely to our expectations we did not find that the size of recall bias was higher in older respondents compared to their younger counterparts. This finding may be explained by participation bias. In our study, response rate was rather low and, possibly, the elderly that did participate may not have been representative of elder trauma patients in the sense that the elderly respondents may experience less memory problems compared to the elderly non-respondents.

We did find that response shift increased with increasing PTSD symptoms. This may indicate that symptoms of PTSD affect cognitive dissonance between the actual health state of the respondent and the desired health state. Second, both TBI and PTSD were positively associated with recall bias. Similarly to TBI, PTSD is associated with impairments in cognitive functioning [36–38]. Our findings clearly confirm that cognitive impairment is a non-trivial factor in research where recall bias may occur.

Agreement between conventional and retrospective change in HRQL was similar to the agreement reported by McPhail et al. and, similarly to McPhail et al., we found a higher retrospective change than conventional change for both the EQ-5D-3 L summary score and EQ-5D-3 L [10]. However, McPhail et al. reported a much larger difference between conventional and retrospective change and higher recall bias. This difference in findings may be explained by the difference in study population and/or timing of HRQL assessments. McPhail et al. studied hospitalized elderly, whereas we studied hospitalized trauma patients aged 18 and older. Higher age of the respondents may have contributed to the difference in conventional and retrospective change and magnitude of recall bias, although our study did not show important differences between conventional and retrospective change, recall bias or response shift in younger versus older trauma patients. With regards to the timing of HRQL assessment, McPhail et al. measured HRQL immediately after hospital admission (T1) and immediately after discharge (T2), whereas our first measurement of HRQL was 1 week post-injury. As a result, the change in HRQL at T1 and T2, as measured by McPhail et al., may have been much larger and subsequently also the contribution of recall bias and response shift to the difference between conventional and retrospective change in HRQL.

### Strengths and limitations

Strengths of our study were the meticulous protocol and the regional coverage, with a high number of respondents. The combined use of subjective and a classification-like scales reinforced analytical opportunities as shown. The high number of respondents allowed us to test for differences between conventional and retrospective change in HRQL and assess recall bias and response shift for specific subgroups of trauma patients, such as trauma patients with severe injury and patients with TBI. The use of the EQ-5D-3 L and EQ-VAS allowed us to compare differences between conventional and retrospective change in HRQL and contribution of recall bias and response shift on a subjective scale and a classification like scale.

A limitation of our study was the uniform follow-up time (3 months), which limited conclusions on duration-dependence of the bias effects. In our study, the time between first and second measurement was 3 months. Stronger recall bias may be expected over longer periods, with perhaps recall becoming more important than response shift [11]. If e.g. 12 months instead of 3 months has been chosen as interval, the effect of recall bias might have been more pronounced.

A second limitation of our study was that the T2 survey included the EQ-5D-3 L and EQ-VAS for the direct measurement of current HRQL as well as the recall and then-test regarding the respondents’ HRQL at T1. This was a challenging task. This meant that the respondents had to fill out several similarly formulated questions. This may have affected both the number of respondents with complete responses, as well as the quality of the responses. Since we administered stand-alone paper-and-pencil surveys, we were not able to verify if the respondents understood the recall and then test and the difference between these two questionnaires.

A third limitation is the use of EQ-5D-3 L rather than the EQ-5D-5 L. As the EQ-5D-5 L has five response options instead of three with more sensitivity and precision, contrasts could have been larger in that case [39–41]. For future studies that aim to investigate recall bias and response shift we recommend to use the EQ-5D-5 L.

### Implications for clinical practice

The findings of our study confirm that, in our sample of trauma patients, there was disagreement between conventional and retrospective change of HRQL and that recall bias and response shift both contributed to this difference. This is important to take into account when change in HRQL is used to evaluate health interventions in this patient group. Whether conventional or retrospective change should be used for the evaluation depends strongly on the aims of the intervention and the characteristics of the patients, as well as which perspective of change is the most important to various stakeholders, as was also pointed out by McPhail et al. [10].

## Conclusions

We conclude that, compared to recall bias, response shift contributed more to the disagreement between conventional and retrospective change in EQ-5D-3 L summary score and EQ-VAS. Predictable subgroups of trauma patients were more susceptible to recall bias and response shift, such as patients who sustained TBI and patients with PTSD symptoms 3 months post-injury.

## Data Availability

The datasets used and/or analysed during the current study are available from the corresponding author on reasonable request.

## Abbreviations

AIS: Abbreviated Injury Scale
ANOVA: One-way analysis of variance
BIOS: Brabant Injury Outcome Surveillance
ED: Emergency Department
HRQL: Health-related quality of life
ICC: Intraclass correlation coefficient
ICU: Intensive Care unit
IES: Impact of event scale
ISS: Injury Severity Score
IQR: Interquartile range
PTSD: Posttraumatic stress disorder
SD: Standard deviation
T1: Postal questionnaire 1 week after the initial treatment of the injury
T2: Postal questionnaire 3 months after the initial treatment of the injury
TBI: Traumatic brain injury
VAS: Visual analogue scale
